# Evaluation of the Efficiency of Two Different Freezing Media and Two Different Protocols to Preserve Human Spermatozoa from Cryoinjury

**DOI:** 10.1155/2016/6059757

**Published:** 2016-07-26

**Authors:** Gemma Fabozzi, Maria Flavia Starita, Emilia Rega, Alessandra Alteri, Antonio Colicchia, Claudio Piscitelli, Pierluigi Giannini

**Affiliations:** FertiClinic, Casa di Cura Villa Margherita, Viale di Villa Massimo 48, 00161 Rome, Italy

## Abstract

It is universally recognized that cryopreservation impairs sperm quality. In order to improve postthawing sperm survival and motility, media of different composition and different protocols have been proposed. However, no clear evidence is available to understand which are the most efficient protocol and medium for sperm cryopreservation. The present study evaluates the efficiency of two different cryopreservation protocols and two common freezing media (FM) containing different cryoprotectants (CPs), TEST Yolk Buffer (TYB) and Sperm Freeze (SF), to preserve human sperm quality. Our data suggest that TYB is better than SF both in terms of postthaw viability and in terms of progressive motility, while the direct addition of FM to the sperm sample resulted in the most efficient protocol in terms of postthaw viability but not in terms of progressive motility.

## 1. Introduction

Sperm cryopreservation has long been used in the clinical practice of assisted reproduction to manage male infertility and to store donor samples or as a tool in the livestock industry. The main goal of male gamete cryopreservation is to preserve sperm viability, motility, and fertilizing ability; however, it has been largely reported in the literature that the freezing-thawing procedures cause severe structural and functional damage to spermatozoa, impairing cell membranes [1–3] and sperm motility [4, 5]. This is due to physical stresses to which cells are usually exposed during freezing: the direct effects of reduced temperature and physical changes associated with ice formation [6]. Furthermore, sperm membrane composition differs from the other cell membranes in the relative proportion of lipid species: there is high proportion of ether-linked fatty acids and phospholipidic hexaenoic acid side chains which contribute to increasing membrane fluidity, while sterols like cholesterol counteract this potential instability [7, 8]. At physiological temperatures, sperm membranes coexist in two phases, fluid and gel, but as the temperature is lowered, a phase transition occurs in favor of the gel form [9]. When cryopreservation is performed, this phase transition leads to a reduction in membrane fluidity, which has been associated with lower sperm survival and motility [10]. Cryoprotectants are hyperosmotic additives which preserve cells by stabilizing intracellular proteins by reducing intracellular ice formation and by moderating the effect of concentrated intracellular and extracellular electrolytes [11]. The first cryoprotectant to cryopreserved spermatozoa was glycerol [12] and it remains the most widely used, generally at a concentration of 10–15% [10, 13–15]. However, other cryoprotectant media have been formulated, such as TEST Yolk and HSPM, both of which contain glycine and glucose [15, 16].

Different protocols for sperm cryopreservation have been defined to improve sperm survival after thawing. Most of them foresee sperm washing prior to freezing; however, Grizard and colleagues [17] reported that the presence of seminal plasma in the cryopreservation medium improved sperm survival. Some groups have suggested different strategies to improve sperm quality when freezing-thawing is performed. For example, Esteves and colleagues [18] speculated that the removal of immotile and damaged sperm prior to cryopreservation may be beneficial, so they used the swim-up method prior to freezing to select a subpopulation of highly motile sperm. Oehninger and colleagues [19] proposed the use of stimulants like pentoxifylline to increase motility.

Although different studies concerning semen cryopreservation are reported in the literature, no clear evidence is available to understand which are the most efficient protocol and FM for sperm cryopreservation. The aim of this study was to evaluate the efficiency of two common FM, TEST Yolk Buffer (TYB) and Sperm Freeze (SF), and two different freezing protocols, one entailing a sperm washing procedure and one not, to preserve sperm quality in terms of cryosurvival and postthaw motility.

## 2. Materials and Methods

### 2.1. Study Design

**Figure 1:**
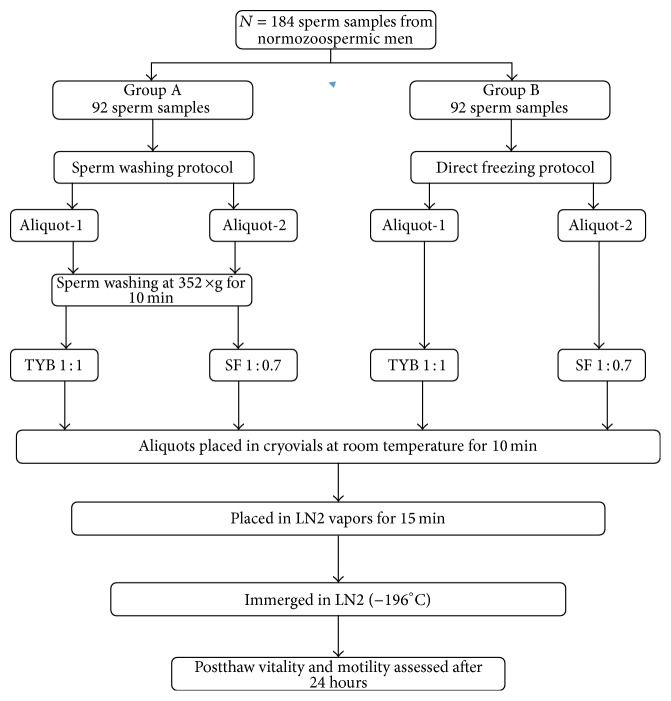
Flow chart of study design.

This is a prospective cohort study. Semen samples were obtained from 184 normozoospermic patients following sperm evaluation performed at FertiClinic, Villa Margherita, from September to December 2014. Patients were randomized in two groups: group A (*N* = 92 samples) and group B (*N* = 92 samples). Samples in group A were split into 2 aliquots and cryopreserved adding, respectively, TEST Yolk Buffer (Irvine Scientific, California) and Sperm Freeze (FertiPro, Belgium) after washing and resuspension. On the contrary, group B samples were split into 2 aliquots and cryopreserved adding, respectively, TYB and SF directly to the semen (Figure 1). Sperm viability and progressive motility were the outcome measures assessed after thawing by a blinded observer. A single team of biologists coordinated all biological work, ensuring that both freezing protocols and viability and motility assessment were standardized.

### 2.2. Semen Analysis

Sperm samples were collected by masturbation after 3–5 days of sexual abstinence and examined by microscopy at 40x magnification after liquefaction. Sperm concentration was assessed using a Makler counting chamber; motility was assessed according to World Health Organization criteria 2010 [20] both before and after cryopreservation.

### 2.3. Comparison between the Two Cryopreservation Methods

In group A, 1 mL of the sperm sample was divided into two aliquots of 0.5 mL and placed into two different tubes containing 4 mL sperm washing medium (sperm washing protocol). Aliquots were then centrifuged at 352 ×g for 10 minutes and each sperm pellet was resuspended with 0.5 mL of HEPES; then, TYB was added to aliquot A1 at a final ratio of 1 : 1 (v/v), while SF was added dropwise to aliquot A2 at a final ratio of 1 : 0.7 (v/v).

In group B, 1 mL of the sperm sample was divided into two aliquots of 0.5 mL and placed into two different tubes; then, FM were added directly to the aliquots (direct freezing protocol): TYB was added to A1 at a final ratio of 1 : 1 (v/v), while SF was added to aliquot A2 at a final ratio of 1 : 0.7 (v/v).

In both cases, FM were added in a dropwise manner, gently mixed, and then placed into cryovials at room temperature for 10 minutes to allow for proper equilibration between the cells and the medium. Then, samples were placed in nitrogen vapors (between −80°C and −100°C) for 15 minutes and finally immerged in liquid nitrogen at −196°C.

### 2.4. Thawing Procedure

The cryopreserved aliquots from both groups were thawed in an identical manner. The cryovial was kept at RT for 10 minutes; then freezing medium was removed by adding 4 mL of sperm washing medium washing the sample in the centrifuge at 352 ×g for 10 minutes. Subsequently, the sperm pellet was resuspended with 250 *μ*L of HEPES: 50 *μ*L was immediately processed for viability, while the rest of the sample was left at 37°C and sperm motility was assessed after 30 minutes by a blinded observer at 37°C.

### 2.5. Viability Assessment

Sperm postthaw viability was assessed using the VitalScreen*™* kit (FertiPro, Belgium) which uses the eosin-nigrosin staining technique to establish the percentage of live spermatozoa assessing the membrane integrity of the cells [20]. The technique is based on the principle that dead cells absorb eosin and as a result stain red. The nigrosin provides a dark background which makes it easier to assess the slides. Briefly, a 50 *μ*L drop of thawed semen was mixed with two drops of 1% eosin stain. After 30 s, three drops of 5% nigrosin were added and the solutions mixed. A 10 *μ*L drop of this mixture was placed onto a microscope slide and a smear was made. The smears were allowed to air dry and were viewed under oil immersion with light microscopy at a magnification of 100x. Live sperm appeared white, while dead sperm with disrupted membranes had absorbed the eosin stain and appeared red. Sperm viability was quantified according to WHO guidelines by counting 200 sperm and expressed as percentage live sperm.

### 2.6. Ethics

All participants enrolled in the study filled out a written informed consent in which they expressed that they consent to donate their sperm for research purposes. The FM and protocols used to preserve sperm samples in this study are routinely used by different IVF units all over the world, so institutional review board was not required.

### 2.7. Statistics

Paired two-tailed Student's *t*-test was used to compare the two FM and the unpaired Student's *t*-test was used to compare the two cryopreservation protocols. Results are given as mean and standard error of the mean. Differences were considered significant at *P* < 0.05. Stepwise linear regression (exit value, *P* = 0.1) was performed to adjust for male age and sperm quality (volume, concentration, morphology, and motility) as possible confounding factors when the two cryopreservation protocols were compared. *β*-regression and the 95% confidence interval (CI) were estimated. SPSS Statistic 21 software was used to perform statistical analysis.

## 3. Results

Semen samples cryopreserved with TYB resulted in better postthaw viability with respect to SF when aliquots were washed and resuspended (Group A) or for the aliquots cryopreserved without washing (Group B). Samples cryopreserved using TYB also showed a better progressive motility in both groups (Table 1).

Then, the freezing protocol was investigated. Sperm viability was significantly higher for samples cryopreserved using direct freezing protocol (Group B) either when TYB was used as freezing medium or when SF was used (Table 1). This result was confirmed even when adjusted for male age and sperm quality, independently from the FM used (*β* = 5.66, CI 95%: 1.8–9.5, and *P* = 0.004 for TYB and *β* = 5.8, CI 95%: 2.9–8.8, and *P* < 0.001 for SF). No significant difference was detected with regard to postthaw progressive motility comparing the freezing protocols either when TYB was used or when SF was used (Table 1). The average motility % change is reported in Table 2.

## 4. Discussion

The aim of this study was to compare the efficiency of two freezing media and two cryopreservation methods to protect sperm quality in terms of cryosurvival and postthaw motility. It is well known that cryoprotectants contained in FM protect cells from cryodamage; however, their toxicity is largely unknown [21, 22]. Stanic and colleagues [21] reported that most of the decrease in sperm motility during cryopreservation can be due to exposure to CPs rather than to the freezing process. Thus, the choice of FM represents a crucial point in cryopreservation procedures. Our results showed significant postthaw viability and progressive motility for samples cryopreserved using TYB with respect to SF, independently from the cryopreservation method used. Our findings are consistent with other studies demonstrating that TYB results in better recovery of motile sperm [21, 23–25]; however, we also investigated the effect on postthaw viability and provided a larger sample size.

The mechanism by which TYB functions is unclear. Each freezing medium is composed by a combination of penetrating osmolytes, which stabilize intracellular proteins, reduce the temperature at which intracellular ice forms, and decrease the impact of intracellular and extracellular electrolytes inside the cell, and nonpenetrating osmolytes, which act as osmotic buffers to protect against cell swelling during the addition/removal of cryoprotectants [22]. Since TYB and SF are composed of the same permeating cryoprotectants (glycerol), probably the nonpermeating component makes the difference: SF contains albumin, while TYB contains egg yolk, which is mainly composed of lipoproteins, phospholipids, and cholesterol. Probably, the low-density lipoprotein fraction of egg yolk, rich in phospholipids, allows for the maintenance of major sperm membrane fluidity, since it permits a greater lipid exchange and contrasts the physiological phase transition in favor of the gel form which normally occurs when the temperature is lowered [9]. On the contrary, albumin contained in SF probably does not succeed in maintaining such fluidity, leading to a lower sperm motility and survival after thawing [10]. Thus, the low-density lipoprotein fraction of egg yolk present in TYB probably exerts a more powerful effect than albumin present in SF, acting as osmotic buffers and protecting the cell against cold shock injury.

The second part of our study was aimed at comparing the ability of two different cryopreservation methods in preserving sperm viability and motility. Data showed that the direct addition of FM to sperm samples resulted in the most efficient strategy in terms of postthaw viability, independently from the FM used. This result can be addressed to the presence of seminal plasma (SP). Its components, in fact, have been shown to be important modulators of sperm function. Notably, animal studies have highlighted that SP proteins seem to inhibit capacitation, lipid peroxidation, and cold shock injury [26–28]. Furthermore, it has been shown that SP is able to protect membrane integrity in boar, ram, and bull sperm [29]. In human as well, it seems to reduce the deleterious effects of cryopreservation, as reported by Grizard and collegues [17]; however, their studies were conducted in free-medium biological material which did not offer adequate cryoprotection of samples.

An additional important aspect is that somatic cells are protected from oxidative stress by antioxidants present within their cytoplasm (superoxide dismutase, catalase, glutathione peroxidase, glutathione, vitamin E, and vitamin C), whereas sperm cells lack a potent intracellular ROS defense system [30], since they lose most of their cytoplasm during their maturation process. However, seminal plasma is well endowed with antioxidant buffer capacity as already demonstrated by different groups [31–33] and all the internal natural antioxidant molecules within SP, such as prostasomes, [34], acetylcarnitine/carnitine, or super oxide dismutase and catalase [35], provide the main sperm protection against oxidative stress.

Comparing the ability of the two different cryopreservation methods in preserving sperm viability and motility, no statistically significant difference was observed in terms of progressive motility, independently from the FM used. This result is in contrast with data reported by Petyim and colleagues [36] who observed a statistically significant difference with respect to progressive motility between samples processed for swim-up prior to cryopreservation and samples processed following cryopreservation: progressive motility was higher in the prepreparation group. This difference can be addressed to the fact that our study included only normozoospermic samples, while the study of Petyim and colleagues [36] included semen samples from infertile males and it has been shown that the quality of semen sample can be related to the outcome of cryopreservation. For example, the presence of dead spermatozoa, cellular debris, bacteria, or leukocytes detrimentally affects sperm survival and the fertility potential after thawing through ROS generation process [37]. In such cases, sperm preparation with swim-up technique is indicated before freezing since it displays an advantage in apoptosis prevention and the stress caused by the cryopreservation procedure does not add to damage caused by free oxygen radicals.

According to authors, results obtained in the presented study can be of significant importance for clinical use, since they can provide advice concerning the best strategy to cryopreserve sperm samples. However, since some studies have shown the seminal plasma of infertile men to have an impaired nonenzymatic antioxidant capacity [31, 32] and others found an erratic distribution between the fertile and nonfertile populations [33], a new prospective study with the same design as this but enrolling pathological samples is in progress in our laboratory. The aim is to obtain a wider range of data and to understand if sperm freezing in the presence of seminal plasma has to be preferred also for these patients or if sperm preparation before freezing or media supplementation with antioxidants can be necessary in such cases. Further researches are always required to improve our knowledge and, in this case, to hopefully yield a universal technique for the cryopreservation of spermatozoa.

## Figures and Tables

**Table 1:** Postthaw viability and progressive motility of semen samples cryopreserved by two different freezing protocols using two different FM.

Variable	Group A (sperm washing protocol)		Group B (direct freezing protocol)		*P* value			
	TYB	SF	TYB	SF	*P* ^a^	*P* ^b^	*P* ^c^	*P* ^d^
Viability (%)	21.20 ± 1	15.62 ± 0.71	27.21 ± 1.69	21.71 ± 1.60	<0.0001	<0.0001	0.006	0.0002
Progressive motility (%)	18.41 ± 1.03	13.99 ± 0.87	16.63 ± 1.67	13.38 ± 1.46	<0.0001	0.005	NS	NS

Note: values are mean ± SEM. TYB, TEST Yolk Buffer; SF, Sperm Freeze.

*P* ^a^, paired *t*-test for TYB versus SF in group A.

*P* ^b^, paired *t*-test for TYB versus SF in group B.

*P* ^c^, unpaired *t*-test for TYB washed versus TYB not washed.

*P* ^d^, unpaired *t*-test for SF washed versus SF not washed.

**Table 2:** Progressive motility values and % change before and after semen samples cryopreservation using two different freezing protocols and two different FM.

Progressive motility (%)	Group A (sperm washing protocol)		Group B (direct freezing protocol)		*P* value			
	TYB	SF	TYB	SF	*P* ^a^	*P* ^b^	*P* ^c^	*P* ^d^
Before	39.10 ± 13.58		41.08 ± 14.2					
After	18.41 ± 1.03	13.99 ± 0.87	16.63 ± 1.67	13.38 ± 1.46				
% change	52.19 ± 2.76	63.81 ± 2.37	58.44 ± 3.82	65.97 ± 3.66	<0.001	0.0114	NS	NS

Note: values are mean ± SEM. TYB, TEST Yolk Buffer; SF, Sperm Freeze.

*P* ^a^, paired *t*-test for TYB versus SF in group A.

*P* ^b^, paired *t*-test for TYB versus SF in group B.

*P* ^c^, unpaired *t*-test for TYB washed versus TYB not washed.

*P* ^d^, unpaired *t*-test for SF washed versus SF not washed.
